# Characteristic Analysis of Metabolic Profiles of *Polygonatum odoratum* (Mill.) Druce from Different Regions of Guizhou Province Based on Non-Targeted Metabolomics

**DOI:** 10.3390/metabo15110733

**Published:** 2025-11-10

**Authors:** Chaoxuan Liao, Qianqian Yang, Chuanqi Zheng, Fuhai Peng, Junxiao Pang, Na Bao, Dali Sun

**Affiliations:** 1 Guizhou Academy of Testing and Analysis, Guiyang 550014, China; liaochaoxuan@gzata.cn (C.L.); baona@gzata.cn (N.B.); 2 School of Public Health, Guizhou Medical University, Guiyang 561113, China; 3 Qianxinan Prefecture Academy of Agricultural and Forestry Sciences, Xingyi 562400, China; 4 School of Food Science and Engineering, Guiyang University, Guiyang 550005, China

**Keywords:** *Polygonatum odoratum* (Mill.) druce, metabolic pathways, amino acids and their derivatives, sphingolipids

## Abstract

**Background:** To investigate the endogenous metabolites in *Polygonatum odoratum* (Mill.) Druce from different geographical origins within Guizhou Province, the metabolic profiles of samples from 12 regions were analyzed using ultra-high-performance liquid chromatography–tandem mass spectrometry (UHPLC-MS/MS). **Methods:** Multivariate statistical methods including principal component analysis (PCA), hierarchical clustering analysis (HCA), and Kyoto Encyclopedia of Genes and Genomes (KEGG) pathway enrichment analysis were employed to explore the influence of geographical origin on the metabolic composition of *P. odoratum*. **Results:** PCA revealed significant differences among samples from different regions which showed clear clustering patterns in our study, indicating that the growing environment considerably affects the metabolite profiles of *P. odoratum*. A total of 6055 potential metabolites were detected in both positive and negative ion modes. Significantly differential metabolites were then screened based on a fold change (FC) ≥ 2 or ≤0.5 and *p* < 0.05. Comparative analysis was conducted on representative samples from three clustered regions: As, ZYMT, and XY−1. The results indicated that alcohols, nucleotides and their derivatives were the major differential metabolites between AS and ZYMT, and alcohols were the key differential metabolites between AS and XY−1, while ketones and sphingolipids were the most significant differential metabolites between ZYMT and XY−1. KEGG enrichment analysis revealed that the pathways of nucleotide metabolism, amino acid biosynthesis, and aminoacyl-tRNA biosynthesis were notably disturbed, suggesting their crucial roles in the synthesis of differential metabolites in *P. odoratum*. **Conclusions:** These findings demonstrated the notable differences in the metabolite composition of *P. odoratum* from different regions of Guizhou province.

## 1. Introduction

*Polygonatum odoratum* (Mill.) Druce, commonly known as Weirui or Zhujingyu, is widely distributed throughout southern China. As a perennial herb belonging to the genus *Polygonatum* within the subfamily *Bambusoideae* (*Poaceae*), it holds significant value in Traditional Chinese Medicine and dietary therapy, with a long history of application [1]. Research on *P. odoratum* has been progressively evolving from traditional empirical knowledge to the analysis of its endogenous components and mechanistic foundations. However, current studies remain predominantly focused on the isolation and purification of individual active constituents such as polysaccharides and flavonoids, and their correlations with specific pharmacological activities [2,3]. Although these investigations have preliminarily demonstrated the potential of *P. odoratum* in antioxidant, immunomodulatory, and hypoglycemic functions [4,5,6,7], the core principle of TCM lies in multi-component, multi-target synergistic actions. Existing research models have thus far failed to fully elucidate the systematic basis of its efficacy, particularly regarding inter-component interactions and the overall in vivo metabolic fate. Moreover, as a medicinal species with broad distribution, a fundamental question remains unresolved: how does environmental heterogeneity influence the internal metabolic network of *P. odoratum* and thereby shape its medicinal quality and clinical efficacy? Geographical variation in medicinal plants is reflected not only in quantitative differences in specific marker compounds but, more significantly, in the comprehensive reorganization of metabolic profiles and the establishment of distinct metabolic patterns [8,9]. Metabolomics have provided powerful tools for systematically analyzing the impact of geographical factors on the distribution of plant metabolites [10,11]. This approach enables high-throughput, unbiased detection and quantification of small-molecule metabolites, capturing subtle but biologically meaningful fluctuations triggered by environmental stress, developmental stages, or genetic variation. It thus offers an integrated perspective on the physiological and biochemical adaptations of plants to their surroundings. The utility of this methodology has been demonstrated in quality assessment studies of various medicinal plants. For example, Lin et al. [12] applied non-targeted metabolomics to identify clear metabolic distinctions between wild-simulated and cultivated *American ginseng*. Their work not only identified 121 shared metabolites but also revealed 22 discriminatory biomarkers including saponins, steroids, and organic acids that accurately reflected cultivation-induced modulation of secondary metabolism. Similarly, Pan et al. [13] combined transcriptomic and metabolomic analyses to investigate polysaccharide accumulation patterns across different *P. odoratum* strains. They identified 14,194 differentially expressed genes and 80 differential metabolites, constructing a co-regulatory network associated with starch, sucrose, and amino sugar metabolism. These studies collectively underscore the value of metabolomics in deciphering quality formation mechanisms and geographical traceability of medicinal plants. Despite these advances, the application of metabolomics to understand how micro-environmental gradients shape metabolic divergence in *P. odoratum* remained limited. A systematic understanding of how environmental factors fine-tune the metabolic network and ultimately influence medicinal quality was still lacking. 

Guizhou Province, located in southwestern China, is recognized as a biodiversity hotspot due to its distinctive geography and varied climate. The region’s complex topography and microclimatic diversity contribute to significant habitat heterogeneity [14,15]. As a key production area for *P. odoratum*, Guizhou offers a natural gradient of ecological conditions, providing an ideal context for studying environment-driven metabolic differentiation. We hypothesized that *P. odoratum* from different locations within Guizhou would exhibit spatially structured metabolic profiles, shaped by local adaptation to factors such as light, water availability, soil micronutrients, and microbial communities. Such differentiation was expected to involve both primary metabolites reflecting energy and material balance and secondary metabolites, which largely account for pharmacological activity. Therefore, a systematic investigation of the metabolic variation in *P. odoratum* originating from Guizhou was designed not only to clarify the scientific basis for its regional quality but also to empirically validate the traditional concept that “the environment shapes medicinal properties.” To address these questions, this study employed UHPLC-MS/MS-based non-targeted metabolomics, combined with multivariate statistics, to compare *P. odoratum* samples collected from 12 locations in Guizhou. Our objectives were to (1) characterize the metabolic composition and inter-regional differences; (2) identify key differential metabolites and related pathways; and (3) reveal the influence of geographical factors on medicinal quality from a metabolic perspective, thereby supporting quality evaluation, resource development, and standardized cultivation.

## 2. Materials and Methods

### 2.1. Sample Preparation

Mature rhizomes of *P. odoratum* (10 plants per site) that were collected from 12 regions (see Appendix A) were freeze-dried and ground into a fine powder. The metabolite extraction protocol referred to the plant metabolomics method established by Pan et al. [13] Precisely 50 mg of the powdered sample was weighed, and 1200 μL of a pre-cooled (−20 °C) 70% methanol aqueous internal standard extraction solution was added. The mixture was vortexed for 30 min. After centrifugation at 12,000 rpm for 3 min, the supernatant was collected, filtered through a 0.22 μm microporous membrane, and stored in sample vials for UHPLC-MS/MS analysis.

### 2.2. Chromatographic Conditions

An LC-30A ultra-high-performance liquid chromatograph (Shimadzu, Kyoto, Japan) coupled with a TripleTOF 6600+ mass spectrometer (SCIEX, Framingham, MA, USA) was used applied to detect the metabolites. The chromatographic conditions were optimized based on reported methods with adjustments made according to the chromatographic response observed for *P. odoratum* extracts [13]. A waters ACQUITY UPLC HSS T3 (2.1 mm × 100 mm, 1.8 µm) was used to separate the metabolites with mobile phase A of ultrapure water (0.1% formic acid) and mobile phase B of acetonitrile (0.1% formic acid). The column temperature, flow rate, and injection volume were set at 40 °C, 0.4 mL/min, and 4 µL, respectively. The gradient elution program was set as follows: 0–5.0 min: 5% B; 5.0–6.0 min: 65% B; 6.0–7.5 min: 99% B; 7.5–7.6 min: 99% B; 7.6–10.0 min: 5% B.

Mass spectrometric data acquisition was performed in both positive and negative ion modes. The electrospray ionization (ESI) source conditions were set as follows: ion source temperature of 550 °C, ion spray voltage of 5500 V (positive ion mode), −4500 V (negative ion mode), declustering potential of ±60 V, collision energy spread of 15 V, ion release width of 15 V, collision energy of ±30 V, and curtain gas of 35 psi.

### 2.3. Data Analysis

Raw data were converted to mzXML format using ProteoWizard v3.0 (ProteoWizard Software Foundation, Palo Alto, CA, USA). Chromatographic peak extraction was performed with the XCMS program 3.18.0 (The Scripps Research Institute, La Jolla, CA, USA). The extracted peaks were aligned and retention time-corrected. Peaks with a missing rate >50% across sample groups were filtered, and the missing values were imputed using the k-nearest neighbors (KNN) algorithm. Peak areas were corrected using the Support Vector Regression (SVR) method.

The corrected data were organized into a two-dimensional matrix containing precise mass-to-charge ratio (*m*/*z*), retention time, and peak intensity. Metabolite identification was conducted by matching experimental data against an in-house database and public databases (including HMDB, KEGG, and Metlin). For precursor ion matching, a high mass accuracy threshold of 5 ppm was applied for MS1 data, consistent with the performance specifications of the TripleTOF 6600+ system. MS/MS spectra were acquired in the *m*/*z* range of 50–1200, covering the typical mass range of small-molecule metabolites. Metabolite annotations were validated by comparing experimental MS/MS spectra with database records using a scoring algorithm that evaluates mass accuracy, isotopic pattern, and fragmentation pattern similarity. An identification score threshold of 0.5 was applied for confident annotation, following established practices in untargeted metabolomics.

Principal Component Analysis (PCA) was performed using the prcomp function in R (base package) 4.1.2 software (R Foundation for Statistical Computing, Vienna, Austria), and heatmaps were generated using the ComplexHeatmap package. Hierarchical cluster analysis (HCA) was conducted on metabolite accumulation patterns. Differential metabolites were screened using a multi-step statistical approach. First, metabolites with a fold change (FC) ≥2 or ≤0.5 were selected. Second, the statistical significance was assessed using Student’s *t*-test with a raw *p*-value < 0.05. To control the false discovery rate in multiple comparisons, the Benjamini–Hochberg procedure was applied to adjust the *p*-values. Metabolites meeting both the FC threshold and a false discovery rate (FDR) adjusted *p*-value < 0.05 were considered statistically significant for all subsequent analyses. This stringent approach minimizes the risk of false positives while maintaining adequate statistical power. KEGG pathway enrichment analysis was performed on the significantly differential metabolites using the clusterProfiler package in R.

### 2.4. Quality Control Analysis

**Figure 1:**
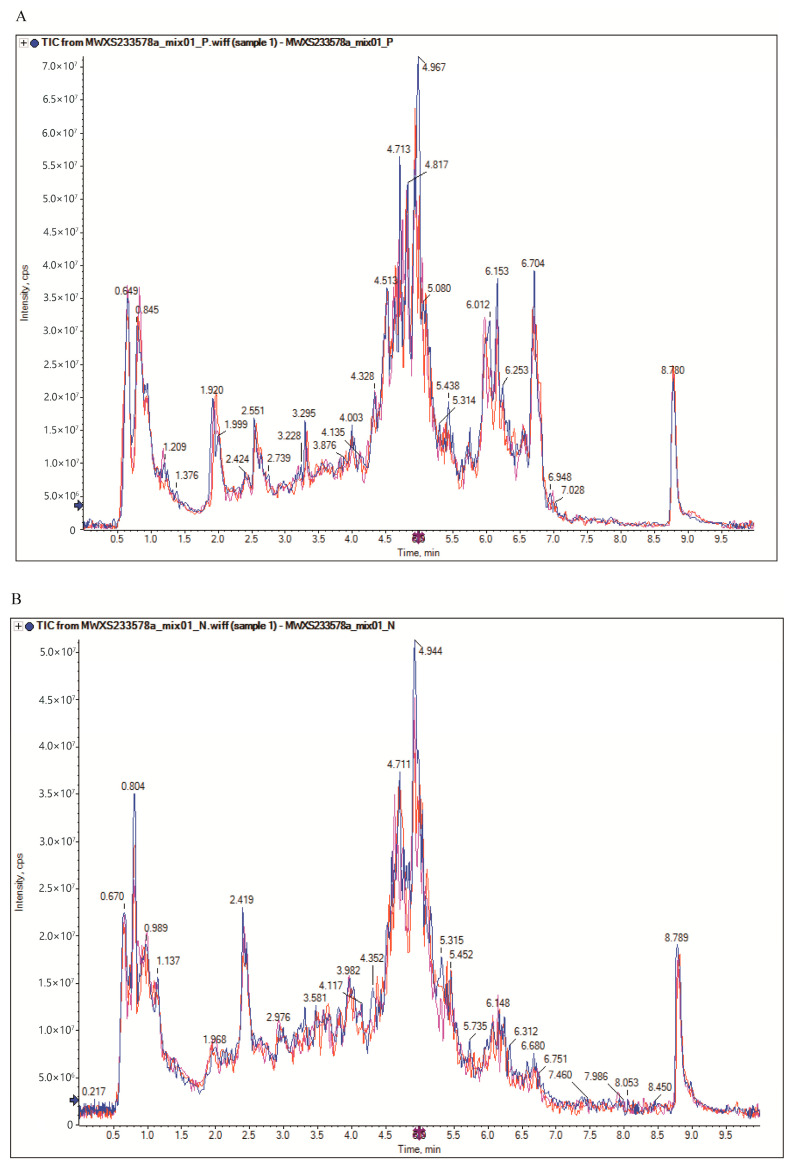
QC sample MS total ion current chromatogram overlay: (**A**) Positive ion mode; (**B**) Negative ion mode. The red, blue, and purple lines represent three independent technical replicates of the pooled QC sample, demonstrating the high reproducibility of the analytical sequence.

To monitor instrument stability, a pooled quality control (QC) sample was prepared by thoroughly mixing 0.50 g of powder from each production area and was then subjected to six replicate analyses by UHPLC-MS/MS. The total ion chromatograms (TIC) of QC samples from mass spectrometric detection were overlaid to evaluate the reproducibility of metabolite extraction and detection. The TIC curves of the QC samples in both positive and negative ion modes showed good overlap (Figure 1), indicating stable instrument conditions and reliable results. Additionally, the PC1 scores of QC samples generally fell within ±3 standard deviations (SD). PCA plots of QC samples, based on ion peaks detected in each sample, showed that all QC samples were within the 3 SD range in both ion modes, confirming no abnormalities and stable instrument performance.

## 3. Results

### 3.1. PCA Results of P. odoratum Samples from 12 Regions in Guizhou Province

**Figure 2:**
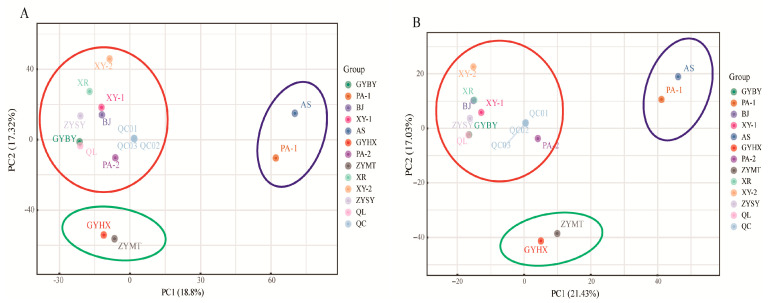
PCA of metabolites from *P. odoratum* samples of different geographical origins: (**A**) Positive ion mode; (**B**) Negative ion mode. The colored circles highlight the three major clusters, which correspond to Cluster 1 (orange), Cluster 2 (purple), and Cluster 3 (green).

PCA was used to analyze the metabolic profiles of *P. odoratum* samples from 12 origins, reflecting inter-group differences and intra-group variability. Figure 2A,B showed the PCA plots in positive and negative ion modes, respectively. In positive ion mode, the cumulative contribution rates of the first and second principal components were 18.80% and 17.32%, respectively. In negative ion mode, they were 21.43% and 17.03%. In both modes, metabolites from different origins clustered into three distinct groups, which were Cluster 1 of XY−1, XY−2, XR, ZYSY, BJ, GYBY, QL, and PA−2, Cluster 2 of PA−1, and AS, and Cluster 3 of GYHX and ZYMT. These results indicated that *P. odoratum* from the 12 origins can be broadly categorized into 3 regions, with significant metabolic differences.

### 3.2. Identification of Metabolite Components in P. odoratum

**Figure 3:**
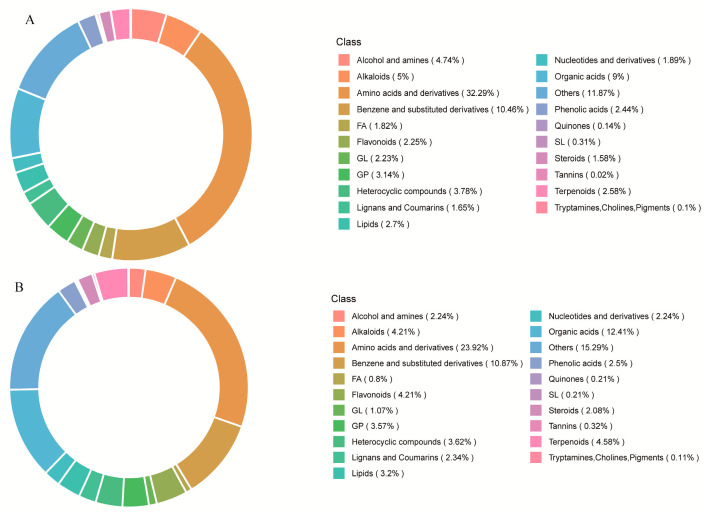
Metabolic composition of *P. odoratum* in (**A**) positive and (**B**) negative ion modes.

Metabolite composition analysis revealed the category distribution of metabolites in *P. odoratum* (Figure 3). In positive ion mode, 4178 metabolites were identified with amino acids and their derivatives being the most abundant (1349, 32.29%), followed by benzene and its substituted derivatives (434, 10.46%). In negative ion mode, 1877 metabolites were identified, with amino acids and their derivatives again predominant (449, 23.92%), followed by organic acids (233, 12.41%). Under both ionization modes, amino acids and their derivatives constituted the predominant categories of metabolites, which indicates that *P*. *odoratum* from Guizhou is rich in these components.

### 3.3. Comparison of Metabolite Components in P. odoratum from Different Regions

**Figure 4:**
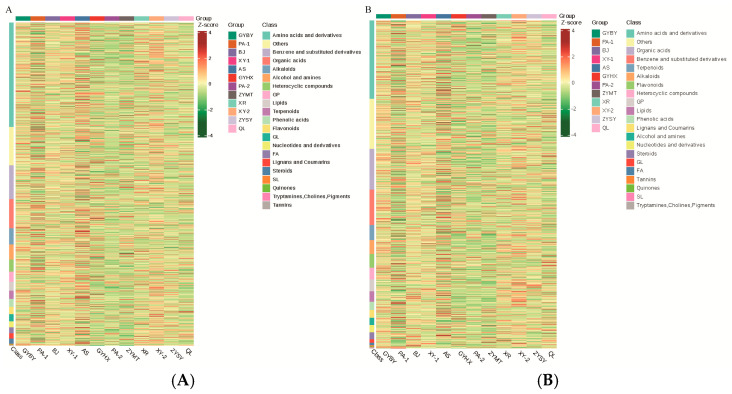
Global clustering of *P. odoratum* metabolites in (**A**) positive and (**B**) negative ion modes. Note: Red indicates high abundance, green indicates low abundance. The left dendrogram represents metabolite clusters, while the top dendrogram shows samples from different geographical regions.

Hierarchical cluster analysis of metabolites from the 12 regions showed significant differences in composition (Figure 4). In positive ion mode, the highest metabolite content in GYBY, BJ, and GYHX was the category of benzene and substituted derivatives, with contents of 3.17%, 3.05%, and 3.16%, respectively. In PA−1, XY−1, XR, XY−2, and QL, amino acids and their derivatives were the most abundant metabolites, with contents of 3.17%, 3.08%, 3.09%, 3.12%, and 3.10%, respectively. In AS, steroids were the most abundant components at 3.17%; in PA−2, organic acids were the highest metabolites, accounting for 3.14%; and in ZYSY, glycerophospholipids were the most abundant component, at 3.07%. In negative ion mode, amino acids and their derivatives were the most abundant in GYBY, BJ, XY−1, GYHX, XR, XY−2, and QL, with respective ratios of 3.09%, 2.88%, 2.97%, 3.11%, 3.02%, 3.08%, and 3.06%. Benzene and substituted derivatives were the most abundant metabolites in PA−1 and ZYMT, accounting for 3.18% and 3.13%, respectively. Steroids, organic acids, and glycerophospholipids were most abundant metabolites in AS (3.16%), PA−2 (3.11%), and ZYSY (2.74%).

Amino acids and their derivatives were the most abundant category, followed by benzene and substituted derivatives and organic acids. In positive ion mode, the highest metabolite content in GYBY, BJ, and GYHX was the category of benzene and substituted derivatives, with contents of 3.17%, 3.05%, and 3.16%, respectively. In PA−1, XY−1, XR, XY−2, and QL, amino acids and their derivatives were the most abundant metabolites, with contents of 3.17%, 3.08%, 3.09%, 3.12%, and 3.10%, respectively. In AS, steroids were the most abundant components at 3.17%; in PA−2, organic acids were the highest metabolites, accounting for 3.14%; and in ZYSY, glycerophospholipids were the most abundant component, at 3.07%. In negative ion mode, amino acids and their derivatives were the most abundant in GYBY, BJ, XY−1, GYHX, XR, XY−2, and QL, with respective ratios of 3.09%, 2.88%, 2.97%, 3.11%, 3.02%, 3.08%, and 3.06%. Benzene and substituted derivatives were the most abundant metabolites in PA−1 and ZYMT, accounting for 3.18% and 3.13%, respectively. Steroids, organic acids, and glycerophospholipids were most abundant metabolites in AS (3.16%), PA−2 (3.11%), and ZYSY (2.74%).

### 3.4. Screening of Differential Metabolites in P. odoratum from Different Regions of Guizhou Province

The precise mass numbers (Q1 mass, mass error ≤ 10^−6^) of metabolites from *P. odoratum* samples were compared with public databases (PubChem, HMDB, Metlin, KEGG). Combined with secondary MS matching scores, 6055 potential metabolites were detected. The top 10 most abundant metabolites were considered characteristic of *P. odoratum* (Table 1). In both ion modes, 12 categories of highly abundant metabolites were identified which were 3 types of amino acids and derivatives, 3 glycerophospholipids, 3 steroids, 2 sphingolipids, 1 lipid, 1 glyceride, 1 organic acid, 1 benzene and derivatives, 1 terpenoid, 1 heterocyclic compound, 1 lignan and coumarin, and 2 others. Among these, three metabolites from GYBY, four from ZYMT, two from QL, two from XY−1, two from XY−2, three from PA−2, one from XR, two from AS, and one from ZYSY.

#### 3.4.1. Analysis of Differential Metabolites in *P. odoratum* from Three Representative Origins

**Figure 5:**
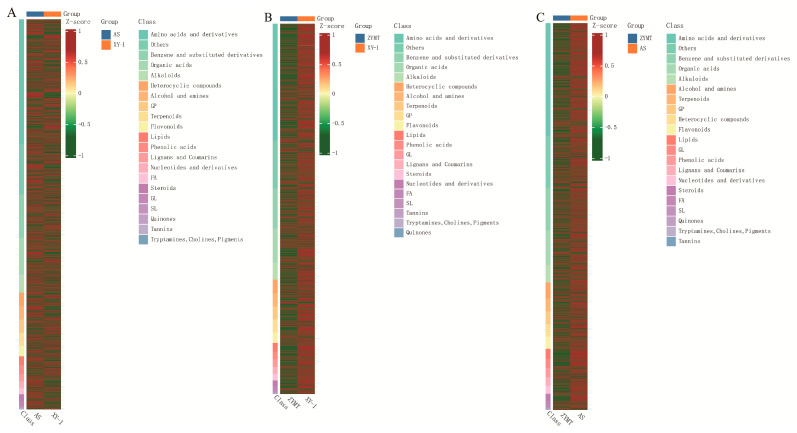
Clustered heatmap of differentially abundant metabolites in *P. odoratum* (**A**) AS vs. XY−1, (**B**) ZYMT vs. XY−1, and (**C**) ZYMT vs. AS.

One representative region from each cluster (XY−1, ZYMT, AS) was selected for pairwise comparison of metabolite differences. Differential metabolites were identified using multivariate and univariate analysis (FC ≥ 2 or ≤0.5) (Figure 5). Amino acids and derivatives, organic acids, and benzene derivatives were the main differential categories, each accounting for >10% of total differential metabolites. Between AS and XY−1, 2573 significant differential metabolites were screened, with 1377 upregulated and 1196 downregulated. Alcohols were identified as the most abundant metabolites. Between ZYMT and XY−1, 2560 differential metabolites were identified, and 66.2% were downregulated. Ketones and sphingolipids were the most abundant metabolites. Between ZYMT and AS, 2774 significant differential metabolites were obtained, with downregulated metabolites accounting for 65.2%. Alcohols and nucleotides and derivatives were the most abundant compounds. Sphingolipids were significantly upregulated in ZYMT compared to other categories, indicating distinct regional characteristics. The total number of differential amino acids and derivatives exceeded 800 in all comparisons, with the highest downregulation proportion (67.4%) in ZYMT vs. AS, indicating significant metabolic differences among the XY−1, ZYMT, and AS regions.

#### 3.4.2. KEGG Enrichment Analysis of Differential Metabolites

**Figure 6:**
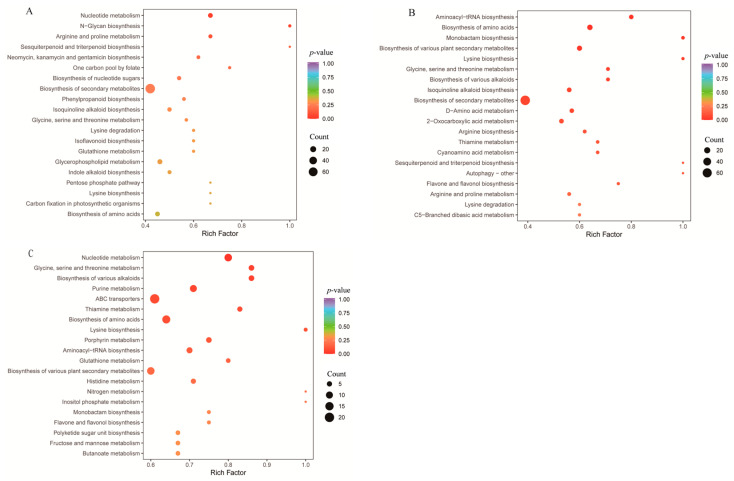
Bubble plot of metabolic pathways (**A**) AS vs. XY−1, (**B**) ZYMT vs. XY−1, (**C**) ZYMT vs. AS.

KEGG pathway enrichment analysis of differential metabolites from AS, XY−1, and ZYMT origins showed that the most significantly different pathway between AS and XY−1 was nucleotide metabolism (*p* < 0.05) (Figure 6). Between ZYMT and XY−1, significant pathways included monobactam biosynthesis, amino acid biosynthesis, aminoacyl-tRNA biosynthesis, biosynthesis of secondary metabolites, and lysine biosynthesis (*p* < 0.05), with amino acid biosynthesis and aminoacyl-tRNA biosynthesis being the most significant pathways (*p* < 0.01). Between ZYMT and AS, differential pathways included glycine, serine, and threonine metabolism, biosynthesis of various alkaloids, and nucleotide metabolism (*p* < 0.05) with nucleotide metabolism being the most significant pathway (*p* < 0.01).

## 4. Discussion

Using a non-targeted metabolomics approach, this study systematically characterized metabolic differences among *P. odoratum* samples from 12 production areas in Guizhou. Metabolite profiling revealed that amino acids and their derivatives, benzene and substituted derivatives, and organic acids were the most abundant classes of metabolites in *P. odoratum*, especially amino acids and their derivatives. This composition provides a material basis for the traditional functions of *P. odoratum* in “strengthening the spleen, nourishing the stomach, and replenishing deficiency.” From a biological standpoint, amino acids serve as fundamental building blocks for proteins and play vital roles in regulation and synthesis, supporting protein formation and cellular stability [16,17]. Their abundance may contribute to nutrient supply, metabolic enhancement, and immune modulation [18]. Additionally, amino acids and derivatives exhibit antioxidant, anti-inflammatory, and antimicrobial properties, aiding in free radical scavenging and oxidative damage mitigation [17,19,20].

Principal component analysis of *P. odoratum* samples from the 12 production areas clearly segregated all samples into three distinct clusters. Cluster 1 comprised XY−1, XY−2, XR, ZYSY, BJ, GYBY, QL, and PA−2; Cluster 2 included PA−1 and AS; and Cluster 3 contained GYHX and ZYMT. Although the three clusters were geographically separated and exhibited variation in mean annual temperature, precipitation, altitude, soil pH, and soil type, these macro-environmental factors did not appear to be the primary drivers of metabolic variation in Guizhou *P. odoratum*. Instead, cultivation site characteristics played a more critical role: Cluster 1 sites were typically sloping fields with moderate soil fertility and good drainage but were drought-prone. Cluster 2 sites consisted of valley terraces with fertile soil, good drainage, and reduced drought stress; and Cluster 3 sites were fertile flat farmland with low drought risk but relatively poor drainage. We therefore propose that soil fertility, water availability, and drainage capacity are key factors driving the differentiation of metabolic composition in *P. odoratum*. This finding aligns with previous reports on other medicinal plants, such as *American ginseng* [12] and *Panax notoginseng* [21], further confirming that the growth environment shapes unique chemical fingerprints by influencing plant secondary metabolism. Key differential metabolites among the three representative production areas (XY−1, AS, ZYMT) exhibited specific distribution patterns, suggesting that *P. odoratum* from different regions may possess distinct medicinal advantages. Comparative analysis identified several key differential metabolites across the clusters, including amino acids and their derivatives, alcohols, nucleotides, and sphingolipids. Particularly noteworthy were the pronounced regional accumulation patterns of amino acids and their derivatives in Cluster 2 (AS production area) and sphingolipids in Cluster 3 (ZYMT production area), which identified them as potential chemical markers for discriminating *P. odoratum* origins. By correlating these region-specific metabolites with known biological activities, we can infer their potential impacts on the pharmacological properties of *P. odoratum*. Specifically, the valley terrace environment of Cluster 2 (AS), characterized by fertile soil and stable water supply, likely promotes efficient nitrogen uptake and assimilation, accounting for the significant accumulation of amino acids and their derivatives. As fundamental building blocks for protein synthesis and precursors to crucial bioactive molecules (e.g., glutathione, neurotransmitters), amino acids directly influence nutritional status, immune function, and stress response [16,17,18]. The marked accumulation of amino acids and derivatives in the AS production area suggests that *P. odoratum* cultivated in Guizhou’s valley terraces may hold superior value for “tonifying” applications, making it particularly suitable for conditions such as physical weakness, malnutrition, compromised immunity, and convalescence, by supplying essential nutritional support and raw materials for synthesizing vital substances. In contrast, the flat farmland environment of Cluster 3 (ZYMT), though fertile, exhibits relatively poor drainage, potentially subjecting roots to periodic hypoxic stress. This environmental pressure may activate the plant’s sphingolipid metabolism pathway, as sphingolipids are crucial membrane components and signaling molecules in plant responses to biotic and abiotic stresses. Sphingolipids are a class of bioactive lipids that regulate cell signaling, differentiation, apoptosis, inflammation, and survival [22]. The relative enrichment of sphingolipids in ZYMT, together with literature reports on their roles in inducing intestinal cell apoptosis and immunomodulation [23,24], strongly suggests that *P. odoratum* from Guizhou’s flat farmland areas may hold unique potential for use in adjuvant anti-tumor therapy or the management of immune imbalance-related diseases.

KEGG pathway analysis linked metabolic divergence to underlying biological processes, highlighting nucleotide metabolism and amino acid biosynthesis as the most affected pathways. Nucleotide metabolism supports growth, stress response, and metabolic regulation [25,26], and its prominence in multiple comparisons suggested its role in local environmental adaptation [27]. Amino acid biosynthesis and aminoacyl-tRNA biosynthesis were particularly relevant in the ZYMT vs. XY−1 comparison, reflecting proteomic adjustments to environmental conditions [28,29,30]. Other differential metabolites such as alkaloids, organic acids, and steroids may also have been influenced by nucleotide and amino acid metabolic pathways, given the interconnections between energy metabolism, biosynthesis, and pharmacological activity [31,32,33]. These findings are consistent with earlier work on *P. odoratum* polysaccharide synthesis [13], collectively illustrating how environmental factors regulate key metabolic pathways to influence overall quality.

In summary, this study demonstrated from a metabolomic perspective that geographical origin decisively influences the chemical quality of *P. odoratum*. By integrating metabolic pathway analysis and differential metabolite identification, we systematically elucidated the mechanism whereby environmental factors regulate core metabolic pathways in *P. odoratum*, thereby affecting the accumulation of secondary metabolites. Notably, we revealed that soil fertility, water status, and drainage capacity likely underlie the regulation of amino acid and sphingolipid metabolism, thereby forming the biological basis for the distinct pharmacological activities of *P. odoratum* from different production areas. These findings not only provide insights into the formation mechanism of the “genuine regional drug” (Daodi) property of *P. odoratum* but also establish a theoretical foundation for developing a quality assessment system based on characteristic metabolite profiles. Future research could further integrate soil metabolomics and transcriptomics data to construct a comprehensive regulatory network linking environmental factors to medicinal component synthesis, thereby providing a more robust scientific basis for the precision cultivation and quality improvement of *P. odoratum*.

## 5. Conclusions

Non-targeted metabolomics was used in this study to analyze metabolic differences in *P. odoratum* from different origins in Guizhou. The results showed clear separation among samples from 12 origins, and 6055 potential differential metabolites were detected. The top 10 most abundant metabolites belonged to 12 categories, with amino acids being the most abundant. Pairwise comparisons among AS, ZYMT, and XY−1 revealed that alcohols and nucleotides and their derivatives were the major differential metabolites between AS and ZYMT. Alcohols were the potential biomarkers between AS and XY−1 and ketones and sphingolipids were the most significant metabolites between ZYMT and XY−1. KEGG enrichment analysis identified nucleotide metabolism, amino acid biosynthesis, and aminoacyl-tRNA biosynthesis as the most significantly different pathways. The differences in metabolite composition may be related to geographical location and climatic conditions. Analyzing differential metabolites from 12 regions helps elucidate the specific bioactive components and medicinal value of *P. odoratum* within Guizhou, providing a scientific basis for drug development, health product applications, and quality evaluation. This study also offers important insights for the development and optimization of germplasm resources and quality assessment of *P. odoratum* in Guizhou, promoting sustained economic growth and sustainable development in the region.

## Figures and Tables

**Table 1:** Information on the top 10 major metabolites in *P. odoratum*.

Metabolite Category	Retention Time (Min)	Adduct	Mass Error (×10^−6^)	Q1 Mass (Da)	Molecular Weight (Da)	Score	Database ID	Formula	Compound	Region with Maximum Content
Amino acids and derivatives	3.33	[M+]^+^	20.40	658.36	658.38	0.67	Metlin (264109)	C_32_H_50_N_8_O_7_	His-Leu-Lys-Tyr-Val	XY−2
	2.07	[M+CH_3_COO]^−^	2.00	521.21	462.17	0.90	Metlin (228141)	C_16_H_26_N_6_O_10_	Ser-Asn-Gln-Asp	AS
	5.00	[M+CH_3_COO]^−^	9.44	519.26	460.25	0.74	Metlin (178582)	C_20_H_36_N_4_O_8_	Leu-Ile-Ser-Glu	ZYMT
GP	4.72	[M+Na]^+^	15.24	1065.55	1042.58	0.73	PubChem (53480149) (HMDB0009954)	C_50_H_92_O_18_P_2_	PIP (18:0/20:3 (5Z,8Z,11Z))	QL
	6.74	[M+H-H_2_O]^+^	1.06	520.34	537.34	0.87	PubChem (16759367)	C_26_H_52_NO_8_P	PC (16:0/2:0)	XR
	6.40	[M-H]^−^	9.90	515.24	727.55	0.63	PubChem (53479648) (HMDB0009050)	C_41_H_78_NO_7_P	PE (18:1 (11Z)/P-18:1 (11Z))	ZYSY
Steroids	4.57	[M-H]^−^	0.15	1095.53	1096.53	1.00	PubChem (85125467) (HMDB0033401)	C_51_H_84_O_25_	Yayoisaponin C	PA−2
	4.66	[M+H]^+^	3.03	917.47	916.47	0.93	PubChem (85137950) (HMDB0033353)	C_45_H_72_O_19_	Agavasaponin C	ZYMT
	4.95	[M+H-H_2_O]^+^	0.74	903.49	920.50	0.65	PubChem (102482481)	C_45_H_76_O_19_	Timosaponin BII	ZYMT
SL	8.62	[M+Na]^+^	22.23	696.54	673.54	0.67	PubChem (5283584) (HMDB0010702)	C_38_H_76_NO_6_P	CerP (d18:1/20:0)	GYBY
	8.74	[M+Na]^+^	3.06	736.53	713.54	0.93	PubChem (10169092) Metlin (85015)	C_40_H_75_NO_9_	Glucosylceramide	GYBY
Lipids	6.01	[M+NH_4_]^+^	0.77	318.30	300.27	0.70	PubChem (12520) KEGG (C03195)	C_18_H_36_O_3_	(R)-10-hydroxystearic acid	QL
GL	8.56	[M+NH_4_]^+^	2.84	792.56	774.53	0.93	PubChem (21582567)	C_45_H_74_O_10_	[(2S)-2-[(9Z,12Z,15Z)-octadeca-9,12,15-trienoyl]oxy-3-[(2R,3R,4S,5R,6R)-3,4,5-trihydroxy-6-(hydroxymethyl)oxan-2-yl]oxypropyl] (9Z,12Z,15Z)-octadeca-9,12,15-trienoate	GYBY
Organic acids	1.10	[M-H]^−^	0.89	133.02	134.02	0.99	PubChem (92824) (HMDB0031518) KEGG (C00497)	C_4_H_6_O_5_	D-Malic acid	XY−1
Benzene and substituted derivatives	2.43	[M-H]^−^	0.02	475.15	476.15	1.00	PubChem (3038513)	C_20_H_28_O_13_	Primeverin	AS
Terpenoids	4.65	[M-H]^−^	13.93	1065.52	1066.54	0.75	PubChem (131751714) (HMDB0035343)	C_54_H_82_O_21_	TR-saponin C	PA−2
Heterocyclic compounds	4.96	[M-H]^−^	1.00	919.49	920.49	0.98	PubChem (131751196) (HMDB0031838)	C_45_H_76_O_19_	Asparagoside E	ZYMT

## Data Availability

Data are contained within the article.

## Abbreviations

The following abbreviations are used in this manuscript:

AS	Pingba, Anshun City
BJ	Qianxi, Bijie City
CV	Coefficient of Variation
ESI	Electrospray Ionization
FC	fold change
FDR	False Discovery Rate
GYBY	Baiyun, Guiyang City
GYHX	Huaxi, Guiyang City
HCA	hierarchical clustering analysis
KEGG	Kyoto Encyclopedia of Genes and Genomes
KNN	k-Nearest Neighbors
PA−1	Xidiantunjiaohe, Pu‘an County
PA−2	Xidianlianglukou, Pu‘an County
PCA	principal component analysis
QC	Quality Control
QL	Dijiulaozhaihe, Qinglong County
SVR	Support Vector Regression
TIC	Total Ion Chromatogram
UHPLC	Ultra-High-Performance Liquid Chromatography
XR	Jiangjiawan, Xingren City
XY−1	Muzhijidi, Xingyi City
XY−2	Niubangzi, Xingyi City
ZYMT	Meitan, Zunyi City
ZYSY	Suiyang, Zunyi City

## Appendix A

**Table A1:** List of the 12 Production Areas for *P. odoratum*.

Code	Production Area	Cultivation Band Type	Soil Type	Soil Fertility	Altitude	Regional Environment Description
AS	Pingba, Anshun City	Farmland	Paddy soil	Fertile	~1100 m	Riverside; terraced fields
BJ	Qianxi, Bijie City	Dry land	Limestone soil	Moderately fertile	~1300 m	Mountainous area
GYBY	Baiyun, Guiyang City	Dry land	Yellow soil	Moderately fertile	~1200 m	Mountainous area
GYHX	Huaxi, Guiyang City	Farmland	Paddy soil	Fertile	~1000 m	Flat terrain
PA−1	Xidiantunjiaohe, Pu‘an County	Farmland	Paddy soil	Fertile	~1450 m	Riverside; terraced fields
PA−2	Xidianlianglukou, Pu‘an County	Dry land	Limestone soil	Moderately fertile	~1500 m	Mountainous area; prone to drought
QL	Dijiulaozhaihe, Qinglong County	Dry land	Limestone soil	Moderately fertile	~1450 m	Mountainous area; prone to drought
XR	Jiangjiawan, Xingren City	Dry land	Yellow soil	Moderately fertile	~1300 m	Mountainous area; prone to drought
XY−1	Muzhijidi, Xingyi City	Dry land	Yellow soil	Moderately fertile	~1300 m	Mountainous area; prone to drought; area with high temperature
XY−2	Niubangzi, Xingyi City	Dry land	Yellow soil	Moderately fertile	~1300 m	Mountainous area; prone to drought; area with high temperature
ZYMT	Meitan, Zunyi City	Farmland	Paddy soil	Fertile	~900 m	Flat terrain
ZYSY	Suiyang, Zunyi City	Dry land	Yellow soil	Moderately fertile	~1000 m	Mountainous area; prone to drought

## References

[1] Zhang Y., Li X., Yu D., Yang Z., Shen Z., Meng Y., Ding Y., Li Y. (2025). Botany, chemistry, bio-activity, and application of *Polygonatum odoratum* (Mill.) Druce: A comprehensive review. Naunyn Schmiedebergs Arch. Pharmacol..

[2] Zhao P., Zhou H., Zhao C., Li X., Wang Y., Wang Y., Huang L., Gao W. (2019). Purification, characterization and immunomodulatory activity of fructans from *Polygonatum odoratum* and *P. cyrtonema*. Carbohydr. Polym..

[3] Li L., Ren F., Chen S., Gao Y. (2009). New homoisoflavanones from *Polygonatum odoratum* (Mill.) Druce. Yao Xue Xue Bao.

[4] Xia G., Li X., Zhang Z., Jiang Y. (2021). Effect of food processing on the antioxidant activity of flavones from *Polygonatum odoratum* (Mill.) Druce. Open Life Sci..

[5] Ye X., Pi X., Zheng W., Cen Y., Ni J., Xu L., Wu K., Liu W., Li L. (2022). The Methanol Extract of *Polygonatum odoratum* Ameliorates Colitis by Improving Intestinal Short-Chain Fatty Acids and Gas Production to Regulate Microbiota Dysbiosis in Mice. Front. Nutr..

[6] Liu J.R., Chen B.X., Jiang M.T., Cui T.Y., Lv B., Fu Z.F., Li X., Du Y.D., Guo J.H., Zhong X.Q. (2023). *Polygonatum odoratum* polysaccharide attenuates lipopolysaccharide-induced lung injury in mice by regulating gut microbiota. Food Sci. Nutr..

[7] Deng Y., He K., Ye X., Chen X., Huang J., Li X., Yuan L., Jin Y., Jin Q., Li P. (2012). Saponin rich fractions from *Polygonatum odoratum* (Mill.) Druce with more potential hypoglycemic effects. J. Ethnopharmacol..

[8] Xu X., Zhu T., Shi T., Chen J., Jin L. (2020). Quality suitability regionalization analysis of *Angelica sinensis* in Gansu, China. PLoS ONE.

[9] Zhang C., Yang D., Liang Z., Liu J., Yan K., Zhu Y., Yang S. (2019). Climatic factors control the geospatial distribution of active ingredients in *Salvia miltiorrhiza* Bunge in China. Sci. Rep..

[10] Jiang M., Peng M., Li Y., Li G., Li X., Zhuang L. (2024). Quality evaluation of four *Ferula* plants and identification of their key volatiles based on non-targeted metabolomics. Front. Plant Sci..

[11] Liu W., Song Q., Cao Y., Xie N., Li Z., Jiang Y., Zheng J., Tu P., Song Y., Li J. (2019). From 1H NMR-based non-targeted to LC-MS-based targeted metabolomics strategy for in-depth chemome comparisons among four *Cistanche* species. J. Pharm. Biomed. Anal..

[12] Lin H., Zhu H., Tan J., Wang H., Dong Q., Wu F., Liu Y., Li P., Liu J. (2019). Non-Targeted Metabolomic Analysis of Methanolic Extracts of Wild-Simulated and Field-Grown American Ginseng. Molecules.

[13] Pan G., Jin J., Liu H., Zhong C., Xie J., Qin Y., Zhang S. (2024). Integrative analysis of the transcriptome and metabolome provides insights into polysaccharide accumulation in *Polygonatum odoratum* (Mill.) Druce rhizome. PeerJ Comput. Sci..

[14] Zhang Z., Huang X., Zhou Y., Zhang J., Zhang X. (2019). Discrepancies in Karst Soil Organic Carbon in Southwest China for Different Land Use Patterns: A Case Study of Guizhou Province. Int. J. Environ. Res. Public Health.

[15] He J., Yan Y.J., Yi X.S., Wang Y., Dai Q.H. (2021). Soil heterogeneity and its interaction with plants in karst areas. Ying Yong Sheng Tai Xue Bao.

[16] Naz S., Liu P., Farooq U., Ma H. (2023). Insight into de-regulation of amino acid feedback inhibition: A focus on structure analysis method. Microb. Cell Factories.

[17] Yang L., Chu Z., Liu M., Zou Q., Li J., Liu Q., Wang Y., Wang T., Xiang J., Wang B. (2023). Amino acid metabolism in immune cells: Essential regulators of the effector functions, and promising opportunities to enhance cancer immunotherapy. J. Hematol. Oncol..

[18] Ling Z.N., Jiang Y.F., Ru J.N., Lu J.H., Ding B., Wu J. (2023). Amino acid metabolism in health and disease. Signal Transduct. Target. Ther..

[19] Kelly B., Pearce E.L. (2020). Amino Assets: How Amino Acids Support Immunity. Cell Metab..

[20] Zhang H.Z., Liu D.H., Zhang D.K., Wang Y.H., Li G., Yan G.L., Cao L.J., Xiao X.H., Huang L.Q., Wang J.B. (2016). Quality Assessment of *Panax notoginseng* from Different Regions through the Analysis of Marker Chemicals, Biological Potency and Ecological Factors. PLoS ONE.

[21] Espinoza K.S., Snider A.J. (2024). Therapeutic Potential for Sphingolipids in Inflammatory Bowel Disease and Colorectal Cancer. Cancers.

[22] Gomez-Larrauri A., Larrea-Sebal A., Martín C., Gomez-Muñoz A. (2025). The critical roles of bioactive sphingolipids in inflammation. J. Biol. Chem..

[23] Birt D.F., Merrill A.H., Barnett T., Enkvetchakul B., Pour P.M., Liotta D.C., Geisler V., Menaldino D.S., Schwartzbauer J. (1998). Inhibition of skin carcinomas but not papillomas by sphingosine, N-methylsphingosine, and N-acetylsphingosine. Nutr. Cancer-Int..

[24] Huang S., Jia A., Song W., Hessler G., Meng Y.G., Sun Y., Xu L., Laessle H., Jirschitzka J., Ma S. (2022). Identification and receptor mechanism of TIR-catalyzed small molecules in plant immunity. Science.

[25] Slocum R.D., Mejia Peña C., Liu Z. (2023). Transcriptional reprogramming of nucleotide metabolism in response to altered pyrimidine availability in *Arabidopsis* seedlings. Front. Plant Sci..

[26] Zhu X., Liao J., Xia X., Xiong F., Li Y., Shen J., Wen B., Ma Y., Wang Y., Fang W. (2019). Physiological and iTRAQ-based proteomic analyses reveal the function of exogenous γ-aminobutyric acid (GABA) in improving tea plant (*Camellia sinensis* L.) tolerance at cold temperature. BMC Plant Biol..

[27] Wang L., Li H., Zhao C., Li S., Kong L., Wu W., Kong W., Liu Y., Wei Y., Zhu J. (2017). The inhibition of protein translation mediated by AtGCN1 is essential for cold tolerance in *Arabidopsis thaliana*. Plant Cell Environ..

[28] Mao X., Zhang H., Tian S., Chang X., Jing L. (2010). TaSnRK2.4, an SNF1-type serine/threonine protein kinase of wheat (*Triticum aestivum* L.), confers enhanced multistress tolerance in *Arabidopsis*. J. Exp. Bot..

[29] Ibba M., Söll D. (2001). The renaissance of aminoacyl-tRNA synthesis. EMBO Rep..

[30] Ling J., Reynolds N., Ibba M. (2009). Aminoacyl-tRNA synthesis and translational quality control. Annu. Rev. Microbiol..

[31] Jones D.E., Perez L., Ryan R.O. (2020). 3-Methylglutaric acid in energy metabolism. Clin. Chim. Acta.

[32] Haist G., Sidjimova B., Yankova-Tsvetkova E., Nikolova M., Denev R., Semerdjieva I., Bastida J., Berkov S. (2024). Metabolite profiling and histochemical localization of alkaloids in *Hippeastrum papilio* (Ravena) van Scheepen. J. Plant Physiol..

[33] Bhambhani S., Kondhare K.R., Giri A.P. (2021). Diversity in Chemical Structures and Biological Properties of Plant Alkaloids. Molecules.

